# Scope of anti-stigma programs against Alzheimer’s disease: A scoping review

**DOI:** 10.1371/journal.pmen.0000406

**Published:** 2025-08-18

**Authors:** Sandra Paola Mondragón Bohórquez, Carolina Gutiérrez-López

**Affiliations:** 1 Facultad de Medicina/Programa Doctorado en Salud Pública/Universidad El Bosque, Bogotá, Colombia; 2 Facultad de Medicina/Coordinador de la Línea de Investigación Educación en Salud del Doctorado en Salud Pública/Universidad El Bosque, Bogotá, Colombia; PLOS: Public Library of Science, UNITED KINGDOM OF GREAT BRITAIN AND NORTHERN IRELAND

## Abstract

This study identifies and reports evidence related to key guidelines for intervention programs aimed at reducing social stigma associated with Alzheimer’s Disease. This scoping review followed the methodology of the Joanna Briggs Institute. The databases searched included: SCOPUS, PubMED, Science Direct, Taylor and Francis, Google Scholar, JBI, Prospero, and Cochrane Library. The STROBE statement was used to organize and draft the protocol. Of the 2275 initial studies, 22 articles were identified for the analysis of emerging categories: social stigma assessment, measurement, and intervention strategies. Of the 2275 initial studies, 22 articles were analysed for the purpose of identifying emerging categories: social stigma assessment, measurement and intervention strategies. Programmes against stigma addressed the following aspects: clinical and epidemiological knowledge and information; emotions, care and impact on patients’ lives; general information on the disease; dementia-friendly communities; a multimedia campaign on stigmatising beliefs; digital platforms; support in the arts; knowledge and clinical aspects; bilingual presentations adapted to the culture; and awareness and knowledge about Alzheimer’s disease. The most common strategies were: patient and intergenerational contact, life stories [vignettes], theatre, music, art, adapting content for culture, curriculum work, discussion groups and health education. The evidence suggests that the content of educational programs and interventions should focus on promoting understanding of the disease, working with groups or peers with the same condition, and generating intergenerational contact, affectionate bonds, and emotional expression to reduce social stigma.

## Introduction

Dementia and cognitive impairment resulting from chronic long-term conditions, such as Alzheimer’s disease (AD), significantly impact healthcare systems due to a lack of management and care programs for this population [1,2]. Furthermore, the demographic data of older adults is currently increasing, as it was 10% of the world’s population by 2021, with a global life expectancy of 46–68 years. In 2022, individuals aged 60 and over outnumbered children under five globally. The population of individuals aged 80 and older is projected to triple from 143 million in 2019–426 million in 2050 [3].

Alzheimer’s disease constitutes 60–70% of all global dementia cases, and this escalating trend necessitates intensified attention and assistance from healthcare professionals and caregivers [4]. According to 2022 data, age is a significant risk factor for Alzheimer’s disease, as the risk increases with age: 5.0% for ages 65–74, 13.1% for ages 75–84, and 33.2% for adults aged 85 and older [1].

This review centers on social stigma, identified by Corrigan et al. [5] y Rosin et al. [6] for these authors social stigma as a devaluing attribute that fosters discriminatory behaviors. These behaviors are characterized by stereotypical relationships, affective reactions, and behavioral responses that individuals develop toward people with mental illness.

Along the same lines, Corrigan [7], drawing from the social psychological model of stigma, explains how attitudes that lead to discriminatory behaviors are promoted and maintained. This framework, grounded in attribution theory, helps to elucidate the relationship between cues, stereotypes, affective reactions, and behavioral responses that individuals exhibit toward people with mental illness.

Within this context, public attitudes contributing to stigmatization—such as authoritarianism and benevolence—have been identified. These attitudes foster social distancing from individuals with neuropsychiatric disorders, portraying them as childlike and irresponsible [5].

Such attitudes vary depending on the specific neurocognitive condition being referenced. For instance, in cases of Alzheimer’s-type dementia, there is a tendency to attribute dangerousness, often associated with aggressive behaviors exhibited by some patients. This, in turn, elicits emotional reactions such as fear toward dementia. Pescosolido et al. [8] further report that the assignment of a diagnosis can trigger stereotypes and negative attitudes among both patients and caregivers.

Apart from the progressive cognitive decline associated with Alzheimer’s Disease (AD), social stigma can have negative consequences. It serves as a chronic and acute trigger for exclusion, rejection, guilt, or devaluation from social judgment. This can lead to a reduced quality of life [5,9,10]. Identifying this process in the face of social stigma facilitates the diagnosis and timely treatment of individuals with a disease, enabling patients and caregivers to make informed decisions regarding coping mechanisms [11].

To implement public health actions aimed at reducing stigma, it is essential to recognize it as a complex social phenomenon that manifests across various contexts of power, leading to the formation of social prejudices that trigger discriminatory actions toward patients [5,9]. Moreover, any effort to reduce social stigma must be grounded in a thorough understanding of the key concepts, principles and characteristics that define this phenomenon [8].

Consequently, anti-stigma programs have been implemented since the 1970s, primarily in high-income countries. Nevertheless, in the current era, stigma reduction efforts have not advanced in Latin America and the Caribbean concerning their nature and scope of implementation [12].

Based on previous research [13,14] practical strategies to reduce public stigma towards AD include contact and psychoeducational interventions, as well as health education programs [15] that encourage dialogue among all stakeholders involved in AD management and care [16]. Other researchers [6,12,17,18] noted a lack of public knowledge about the disease and identified the need for anti-stigma strategies to evaluate intervention effectiveness. Simply increasing knowledge and symptom recognition may not necessarily reduce social stigma [5].

Therefore, the review aims to analyze the existing literature to identify the key components of programmes and interventions focused on the reduction of social stigma related to Alzheimer’s disease.

## Methodology

This scoping review was structured according to the Preferred Reporting Items for Systematic reviews and Meta-Analyses extension for Scoping Reviews (PRISMA-ScR)] presentation guidelines proposed by Tricco et al. [19]. The methodology is based on Arskey and O’Malley’s five-step framework: 1. identifying the research question and objectives, 2. structuring the search strategy to identify relevant trials and grey literature, 3. establishing inclusion and exclusion criteria, 4. data collection, and 5. collating, summarising and reporting the results [20]. The STROBE statement was used to organize and draft the protocol. The scoping review protocol was registered on Open Science Framework (https://osf.io/fc28g) under the code osf.io/cr9et, following Joanna Briggs [JBI] guidelines [21]. The first stage of the review entailed formulating the research question using the PCC strategy (P = Population: individuals with Alzheimer’s disease; C = Concept: social stigma; C = Context: programs and interventions at the primary healthcare level). The full electronic search strategy was (“social stigma” OR “family stigma”) AND (“alzheimer disease” OR “alzheimer dementia”) AND (“health education” OR intervention OR “evaluation program”).

Subsequently, we performed term normalization in Health Science Descriptors/Medical Subject Headings (DeCS/MeSH), and its Boolean connectors using the SCOPUS bibliometric database to conduct a literature search for the past 13 years [2011–2024]. The most significant evidence of publications was found in 2011. Technical terms were defined and used consistently throughout the text. Additionally, the following databases were consulted: PubMED, Science Direct, Taylor and Francis, Google Scholar, JBI, Prospero and Cochrane Library.

Grey literature was also consulted from documents obtained from the World Health Organization (WHO), the Pan American Health Organization (PAHO), national sources such as the Ministry of Health and Social Protection (MinSalud), and international documents.

Inclusion criteria focused on articles containing original data analyses, including quantitative, qualitative, and mixed studies and reviews of anti-stigma programs and interventions in AD. Excluded materials were opinion articles, abstracts, book reviews, technical reports, weblogs, newsletters, catalogs of products and services of companies, and other non-scientific materials. Additionally, studies discussing stigma in people with mental illness were excluded.

For the selection and extraction of evidence, the process began with reading the title and abstract, which were reviewed by two reviewers (MS and GC). To eliminate duplicates, the Rayyan software was used [22]. Next, we extracted data from the studies based on the eligibility criteria and by reading the full text. The analysis was conducted independently by two pairs of reviewers, and in cases of disagreement, a comparison of analysis of the reviewed articles was carried out. If any doubts arose, we sought the opinion of a third reviewer who specialized in the area of study (PV).

Although critical appraisal of articles is not mandatory for scoping reviews, it is undertaken to characterize the overall quality of the included articles, as this provides another descriptive indicator of the extent of research in the field of anti-stigma interventions. To determine the quality criteria of the articles, we analyzed them using the Critical Appraisal Skills Programme (CASP) [23] and the Strengthening the Reporting of Observational Studies in Epidemiology Statement (STROBE) [24]. The quality of a study did not determine its inclusion or exclusion from our review (Table 1).

**Table 1:** Critical appraisal within sources of evidence.

Authors	Year	Type of Source	Research Desing	Target population	Conceptual/ theoretical framework	Details inclusion and exclusion criteria	It is methodologically sound	Comprehensiveness of reporting	Reported challenges and limitations
Ministerio de Salud y Protección Social.	2014	Strategy guidelines	Gray literature	N	Y	NA	U	N	N
Kim et al.	2021	Research Article	Randomised controlled trial	Y	Y	Y	Y	Y	Y
Rodrigues y Mathias.	2016	Research Article	Pre experimental research design of one group pretest and posttest	Y	Y	Y	Y	Y	Y
Patel et al.	2021	Research Article	A cross-sectional study	Y	Y	Y	Y	Y	Y
Prins et al.	2020	Research Article	Quantitaive Unclear	U	X	U	Y	Y	Y
Stites et al.	2022	Research Article	Experiment.	Y	Y	Y	Y	Y	Y
Goldman & Trommer	2019	Research Article	Qualitative phenomenology	N	Y	Y	U	Y	Y
Chermahini et al.	2021	Reserach Article	Non-randomized controlled trial	Y	Y	Y	Y	Y	Y
Harris y Caporella	2014	Research Article	Qualitative Phenomenological	Y	Y	Y	Y	Y	Y
Bray et al.	2015	Final Report	Gray literature	Y	Y	Y	NA	NA	N
Bergeron et al.	2021	Research Article	Quasi-experimental	Y	Y	Y	Y	Y	Y
Paone et al.	2015	Final Report	Gray literature	Y	Y	Y	NA	NA	N
ADI & OPS	2019	Strategy guidelines	Gray literature	N	Y	NA	NA	NA	N
Isaacson et al.	2018	Reserach Article	Quantitative Unclear	Y	Y	Y	U	U	Y
Kontos et al.	2020	Reserach Article	Thematic analysis	Y	Y	Y	Y	Y	Y
Burns et al.	2018	Research Article	Quantitaive Unclear	Y	Y	Y	Y	Y	Y
Au et al.	2020	Research Article	Randomised double-blind parallel group trial.	Y	Y	Y	Y	Y	Y
Adams y Cotter.	2011	Final Report	Gray literature	Y	Y	Y	NA	Y	N
Werner y Kermel.	2018	Research Article	Cross‐sectional posttest‐only survey	Y	Y	Y	Y	Y	Y
Perales et al.	2020	Research Article	One arm pre-post trial	Y	Y	Y	Y	Y	Y
Rosenzeweig.	2021	Research	Gray literature	Y	Y	Y	Y	Y	Y
Montepare y Pandolfi.	2022	Research	Quantitative study	Y	Y	Y	U	Y	Y

Note: The letters Y = Yes, N = No, U = Unclear and NA = Not applicable represents the criteria that each data set under analysis has met.

The data from each study were organized in Microsoft Excel 2016 using a bibliographic matrix and the criteria described by Gómez et al.[25] and Joanna Briggs-JBI [26]. Next, the content analytic matrix was employed to map the data [24], allowing for the identification of categories such as nature, distribution, contributions of studies, intervention strategies, and anti-stigma programs.

## Findings

The search strategy identified 2,252 publications across eight databases (110 in PubMed; 99 in Taylor & Francis; 54 in Scopus; 1,780 in Google Scholar; and 201 in ScienceDirect, totalling 2,244 studies) and three studies were found in JBI, one in Prospero and four in the Cochrane Library, totalling eight preliminary studies.

**Fig 1:**
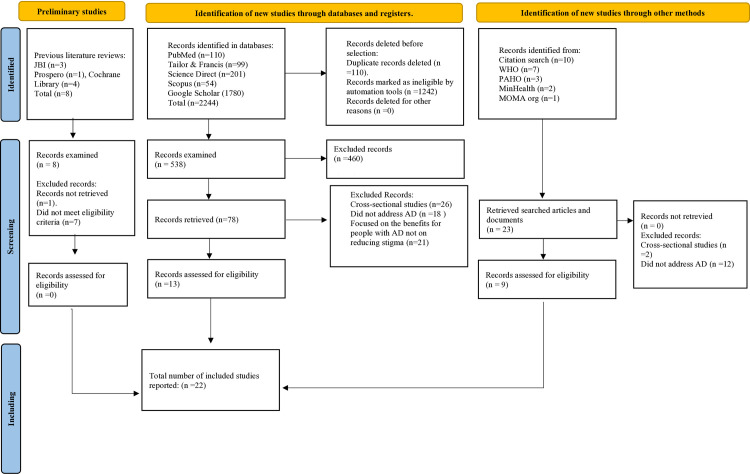
Search flow chart on anti-stigma intervention programmes 2011-2024. Flow chart illustrating the articles and documents included in the study. Taken from Page et al., [27].

Concerning gray literature, we found a total of 23 relevant documents through various sources. Specifically, seven documents were identified on the WHO website, three documents on the PAHO anti-stigma platform, two publications from the National Ministry of Health, one study on the Meet Me - MoMA platform hosted on the Museum of Modern Art of New York’s website, and an additional 10 papers were discovered through cross-data search. Finally, the sum total of the documents included in the review, considering the databases, previous studies and the results of the gray literature search, yielded 2275 articles. (Fig 1) [27].

To select the studies, records from eight databases were imported into Rayyan software [22], resulting in the removal of 110 duplicate articles from a total of 2,244 entries. An additional 1,242 articles were excluded after reviewing the titles and study objectives, as they did not focus on the phenomenon of interest (social stigma in Alzheimer’s disease).

Subsequent screening of 538 records based on abstracts identified 78 articles that met the eligibility criteria. Of these, 65 were excluded for the following reasons: 26 were cross-sectional studies, 18 did not address Alzheimer’s disease, and 21 focused on the benefits for people with Alzheimer’s rather than on stigma reduction.

Finally, eight previously published reviews and 14 documents from the grey literature were excluded, as they did not report implementation data for stigma reduction programs. Among these, two were cross-sectional studies, and 12 did not address Alzheimer’s disease. This resulted in 22 articles being included (Fig 1). According to the proposal of Cochrane Handbook for Systematic Reviews of Interventions Version 5.1.0 [28], the articles were excluded by lack of access to full text or failure to meet inclusion criteria. The analysis of the findings encompasses two primary themes that affect anti-stigma programs, namely the intervention strategies and components of the studies. Additionally, three general themes of the studies described below.

### Characteristics of the studies

Of the 22 studies included in this analysis, 13 employed a quantitative approach, three were qualitative [29–31], and three were intervention reports [32–34]of which two used a mixed-methods design [32,33]. Additionally, two studies focused on program guidelines and proposals [4,35], and one study implemented a digital platform for anti-stigma interventions. All methodological approaches were identified and described.

According to the country of publication, the United States has nine studies, followed by India, and the United Kingdom, each with two studies. Australia, Canada, China, Israel, Iran, Latin America, the Caribbean, the Netherlands, and Colombia each have one article. The table presented in Table 2 provides an overview of the contributions to knowledge and innovation from various studies on Alzheimer’s disease (AD).

**Table 2:** Anti-stigma programs and interventions developed by country and study characteristics.

Country	First Author and Year	Objective	Participants	Type and Design	Tools use to measure	Characteristics of programs
U.S.A	Perales et al. (2020)	Increase knowledge about AD, adapted to the Latino culture and community.	61 latino adults Gender: 37 women, 24 men 75 years old on average	Quantitative pre-experimental	A pre-post-survey ADRD knowledge five-point Likert scale	Through bilingual, culturally appropriate presentations, they develop the Ageing with Dignity programme by addressing disease knowledge and clinical aspects.
U.S.A	Bergeron et al. (2021)	To determine the effectiveness of two AD knowledge interventions in two Afro-American neighbourhoods.	103 participants 55–65 years and over.	Quasi-experimental with a control group.	Alzheimer’s Disease Knowledge Scale (ADKS)	The Dementia Friendly America Kit focuses on knowledge and clinical aspects of the disease.
U.S.A	Goldman & Trommer (2019).	To evaluate the impact of the “A Friend for Rachel” programme on students in the pre-medical programme.	101 medical students	Qualitative	Write weekly reflections about their interactions with their friends living with dementia	There are five main themes: knowledge of dementia, care, emotions experienced, impact on career choices and life, and the impact of dementia on the family.
U.S.A	Harris et al. (2014)	Creation of a dementia-friendly community through an intergenerational choir.	13 patients and one family member; and 13 college students. Age range: 18–72. Gender: prevalence women	Qualitative	Semi-structured open-ended questions on attitudes and knowledge about AD, collected	The focus is on aspects of emotion, care and impact on life.
U.S.A	Isaacson et al.(2018)	Evaluation of the effectiveness of the social networking platform Facebook.com as a tool for the recruitment of subjects to visit AlzU.org.	503 visitors Gender: 79.8% women and 21.2% men. Age range: 30–90 years old.	Quantitative	After completing lesson and activity content, a post-survey assessed Likert-scale ratings on several website usability parameters and user satisfaction.	Provides information on the prevention, treatment and care of Alzheimer’s disease.
U.S.A	Rosenzweig (2021)	To determine the effectiveness of vignettes in reducing stigmatised attitudes to AE.	50 participants, Control group: 22 Intervention group: 28. 65 years and older	Longitudinal Quantitative	Dementia Attitude Scale (DAS) pre/posttest scores in the intervention group	Educating and raising awareness of Alzheimer’s disease.
U.S.A	Stites et al. (2022)	To determine whether preclinical diagnosis, through the use of vignettes, contributes to the reduction of stigma towards Alzheimer’s disease.	1,817 participants recruited over two years.	Experimental 3 × 2 × 2 factorial design	Modified Family Stigma in Alzheimer’s Disease Scale (FS-ADS).	Addressing the impact of Alzheimer’s on life, seeking to reduce stigma
U.S.A	Paone (2015)	Implementation of a free platform for the creation of dementia-friendly communities.	General public.	Mixed	Does nor Report	Impact of AD on lives, support in groups and communities.
U.S.A	Adams y Cotter (2011)	To determine the impact of museum programmes for people with dementia or Alzheimer’s disease. Dementia or AD (MoMA)	66 members of the general public	Cross-sectional Study	Surveys designed online	Life impact, community work and art strategies
China	Au et al. (2020)	It examined the effects of perspective-taking intervention in intergenerational care contexts.	72 caregivers of people with dementia.	Randomised double-blind parallel group trial.	Satisfaction With Life Scale (SWLS) Zarit Burden Interview Center for Epidemiologic Studies Depressive Scale (CES-D)	It focuses on clinical aspects of AD and knowledge.
United Kingdom	Montepare & Pandolfi (2022)	It explored the need to include beneficial educational opportunities in dementia and AD.	106 respondents from the general public.	Quantitative study, Cross. -sectional	Alzheimer’s Disease Knowledge Scale (ADKS)	Awareness, knowledge of the disease and clinical aspects of Alzheimer’s.
United Kingdom	Bray et al. (2015)	Raising awareness of dementia in BAME communities - Alzheimer’s Society Connecting Communities project	1950 participants	Mixed method Report	Questionnaires for organizations, semi-structured interviews, Observations of the sessions	Addressing awareness, knowledge and clinical aspects of Alzheimer’s disease
Israel	Werner et al. (2018)	To investigate the effect of exposure to a multimedia campaign on stigmatising beliefs about Alzheimer’s.	510 Jewish participants aged 40 and over. 51% female	Quantitative Post-test cross-sectional survey	Checklist listing the core strategies based in the intervention manual.	Work on the impact on life, community work and art strategies and beliefs about Alzheimer’s to reduce stigma.
Canada	Kontos et al. (2020)	Determine Cracked programme effectiveness.	Healthcare staff, caregivers, friends of people with dementia and AD, and the general public.	Qualitative	Structured measures were used to assess the main research questions	It addresses life impact, community work and art strategies.
U.S.A	Burns et al. (2018)	Examined the influence of a theater performance on the emotional affect of a general audience	147 participants	Quantitative	Preliminary effectiveness (before and after the performance using three questions	The impact a play in the emotional affect of a general audience.
Australia	Kim et al. (2021)	Evaluation of the short-term feasibility and effectiveness of an online intervention programme..	208 participants, over 40 years old	Randomised controlled trial	Attribution Questionnaire Dementia Knowledge Assessment Scale (DKAS) - version 2	Addressing awareness, knowledge and clinical aspects of Alzheimer’s disease
India	Rodri- gues & Mathias (2016)	Determination of the effect of an education programme on Alzheimer’s disease.	50 relatives. Age range: 31–50 years. Gender: 100% female	Preexperimental research design of one group pretest and posttest	The structured knowledge questionnaire on Alzheimer’s disease prepared by the investigator consisted of 2 sections	The results of the study showed: Relatives of older people lacked knowledge about Alzheimer’s disease.
India	Patel et al. (2021)	To assess whether Awareness raising and training of junior doctors may improve knowledge and awareness of dementia.	82 senior medical students.	Cross-sectional with pre and post	Alzheimer’s Disease Knowledge Scale (ADKS) Dementia Attitude Scale (DAS)	Awareness, knowledge and clinical aspects of Alzheimer’s disease
Iran	Chermahini et al. (2021)	Determine the impacts of an educationalprogramby using group discussion on perceived stigma in family caregivers of people with Alzheimer’s disease (AD).	66 family caregivers	This was a nonequivalent non-randomized controlled trial	Stigma Impact Scale (SIS).	Group discussion about prognosis, the behavioral problems of afflicted personsog AD, stigma and its types, family caregivers’ experiences of stigma, strategies to prevent or reduce stigmatization, coping skills, and strategies to manage tensions and feelings.
Netherlands	Prins et al. (2020)	Evaluation of an online media production called ‘The Alzheimer Experience’ (AlzExp).	213 members of the general public and relatives. Average age: 44 years. Gender: 187 women and 26 men.	Preexperi-mental	Dementia Questionnaire (ADQ) Alzheimer’s Disease. Knowledge Scale (ADKS)	Addresses awareness of Alzheimer’s disease, knowledge and clinical aspects of the disease
Latin America & Caribbean	ADI &OPS (2019)	Let’s Talk About Dementia“dementia and Alzheimer’s awareness campaign for the Americas region.	Public at large	Virtual stage	Does nor Report	Its focus is on awareness, knowledge and clinical aspects of Alzheimer’s disease.
Colombia	MinSalud (2014)	Guidelines for the reduction of the stigma of mental health.	Public at large	Mental Health Stigma Reduction Communication Strategy	Does nor Report	Awareness of AD, knowledge and clinical aspects of the disease

Note: This table presents the main characteristics of the research.

Among the studies that employed quantitative approaches, the intervention topics primarily focused on the following areas: clinical and epidemiological knowledge and information about Alzheimer’s disease (AD) [36–39]; emotional aspects, caregiving, and the impact of AD on patients’ lives [29,40-42]; general information about AD, treatment, and preventive management within the framework of dementia-friendly communities [43]; multimedia campaigns targeting stigmatizing beliefs [44]; programs utilizing free-access digital platforms [45]; arts-based interventions aimed at reducing stigma [30,31,34,46]; clinical knowledge and understanding of the disease through bilingual, culturally adapted presentations [47]; and awareness-raising and educational efforts regarding AD [48,49].

Of the 14 studies, 11 used at least one validated scale to measure outcomes related to emotions and stigmatizing attributions, as well as knowledge of Alzheimer’s disease. These studies reported internal consistency data that varied across instruments. Two studies used non-validated scales that were adapted specifically for the research. Most of the instruments employed had psychometric evidence supporting their adequacy and met at least one of the following criteria: the authors reported a Cronbach’s alpha of 0.71 or higher; the instrument was described as reliable or valid; or there was some form of evidence for validity or reliability as determined by the researchers.

The review included non-randomized studies, seven of which did not include a control group. Six studies incorporated comparisons with control groups [39,41,42,44,45,50], among which four reported statistically significant differences between intervention and control group scores. In contrast, the studies by Kim et al. [39] and Bergeron et al. [42] reported changes between group scores that were not statistically significant.

Most of the quantitative and qualitative studies that examined medium- or long-term outcomes were conducted in high-income countries (66.6%), targeted the general public or students (66.6%), and implemented interventions involving education or information about Alzheimer’s disease (43%). Among the qualitative studies, one article focused on an arts-based program aimed at reducing stigma [33]. Additionally, 33.4% of the studies followed a descriptive approach using thematic analysis, and two addressed issues related to emotions, caregiving, and the impact of AD on patients’ lives [31,32]. Furthermore, 66.6% employed a phenomenological approach to explore the lived experience of stigma. The instruments used included focus groups and semi-structured surveys. The populations targeted were university students and the general public. Regarding reproducibility criteria, these studies were considered feasible and relevant within the sociocultural context to be replicated in specific settings such as the United States and Canada.

Two of the technical reports addressed general information about Alzheimer’s disease, including treatment and preventive management within the development of dementia-friendly communities [30,35], while another focused on the use of museums as tools for raising awareness [49]. All three reports targeted the general public, primary caregivers, and individuals with AD. Only two reports employed mixed-methods approaches for data collection and analysis [30,35]. The documents outlining guidelines for anti-stigma programs provided implementation directives and adaptable materials, but did not report on actual implementation or outcomes [4,48].

Of the total original studies, technical reports, and program guidelines analyzed, approximately 13.6% of the included studies were randomized controlled trials (RCTs), 77% were pre-post studies with or without control groups, and 9.2% were qualitative studies or research reports. Only 27% of the studies included a follow-up assessment between 1 and 6 months after the intervention ended, and none reported long-term follow-up (i.e., one year or more post-intervention). Since most studies included only two initial time-point measurements (pre-post), the ability to assess the long-term effectiveness of the interventions in reducing stigma is limited.

It was also noted that not all studies and technical reports specified the duration or implementation period of the programs [38,45,46,47,48]. Most programs reported short-term implementation periods, typically lasting less than one month. A smaller proportion of studies examined medium- and long-term durations, defined as 1–6 months and more than 6 months, respectively. Among the quantitative studies with pre- and post-intervention measurements, program durations ranged from one week [37,44], to two to six weeks [40,42,43], and eight weeks [34,36]. In contrast, the randomized controlled trials reported intervention durations of two, four, and six weeks [39,41,50].

The qualitative studies reported longer implementation periods for the programs, ranging from 10 to 12 months [31–33]. The longest program durations were found in the technical reports, which indicated timeframes of two to three years [30,35,49]. These findings suggest that programs with follow-up periods of up to three months post-intervention reported sustained changes in knowledge, stigmatizing attitudes, or behaviors toward individuals with Alzheimer’s disease and their caregivers.

Finally, two programs focused on raising awareness and knowledge about Alzheimer’s disease. One implemented educational strategies and life stories targeted at individuals over 60 years of age and included a control group [48]. The other study incorporated education, contact, and curriculum-based interventions directed at university students [49]. These programs reported varied outcomes. The first study found that the change in the total score on the stigma assessment scale was statistically significant, with attitude changes appearing to result primarily from increased knowledge. In contrast, the second study found that students expressed concern about developing AD, which was not associated with their prior experience of having known someone with the disease.

### Regarding intervention strategies and programs to address the stigma of Alzheimer’s disease

Two studies explored programs utilizing easily accessible digital platforms [35,45], which linked the public through social networks and provided tools for sharing concise and accessible information and experiences about AD. Four programs incorporating art (museums, plays, intergenerational choir) [30,31,34,46] were developed using strategies like intergenerational contact and emotional impact through theater. Two studies implemented multimedia campaigns to combat stigmatizing beliefs [44,45]. One study use the focal groups in caregivers [40].

One key strategy identified by some studies is utilizing contact with patients and primary caregivers. These studies reported that implementing such interventions led to noticeable changes in knowledge and negative attitudes toward AD [29,30,38,42].

Some research has focused on working with minority populations by utilizing cultural adaptation and native language to transmit information and content [33,43,44]. In addition, some studies have implemented health education as a primary strategy in undergraduate formative education through symposia [39,49], as well as employing techniques based on adapted and contextualized stories of the day-to-day lives of individuals diagnosed with Alzheimer’s disease [41,48].

Finally, the guidelines proposed to reduce stigma towards mental illness and AD in Colombia address informal and institutional communication, communication for social mobilization, and mass communication through the media, including workshops (Table 3) [4].

**Table 3:** Components of programmes to reduce the social stigma associated with Alzheimer’s disease.

Anti-stigma programs	Authors	Country	Description/ Program components	Strategies identified in studies
Clinico-epidemiological knowledge and information about AD.	Ministry of Health and Social Protection(2014)	Colombia	Communication of AD characteristics at informal and institutional levels. Emphasis on communication for social mobilisation and mass communication through the media.	Using the Mass Media to educate
	Kim et al. (2021)	Australia	Education and contact approach. Online programme (ED), simulated contact with people with dementia and carers (CT), education and contact (ED + CT) and active monitoring.	Education + contact
	Rodrigues y Mathias. (2016)	India	Planned educational programme on Alzheimer’s disease using PowerPoint and graphics for 45 minutes.	Education
	Patel et al. (2021)	India	Interns prepare a 2-hour interactive symposium on important aspects of the epidemiology, risk factors, symptoms, diagnosis and treatment of the disease.	Education + Curriculum
	Prins et al. (2020)	Netherlands	This programme addressed knowledge about dementia, knowledge about the impact of dementia on family and other informal carers, and attitudes towards people with dementia.	Education+Platform
Feeling, caring and impacting on the lives of people with Alzheimer’s disease	Stites et al. (2022)	U.S.A	Use of vignettes (stories that included a variety of clinical symptoms, biomarkers, and options for treatment or non-treatment).	Education + education and life stories
	Goldman & Trommer. (2019)	U.S.A	It proposes contact strategies, incorporates experiential learning, training and mentoring, and provides students with a meaningful and sustained relationship with a person with dementia.	Education + Contact + Intergenerational Interaction
	Chermahini et al. (2021)	Iran	Discussion groups to share stories and experiences of caring, discussion about Alzheimer’s disease, prognosis, behaviour, problems of people with the diagnosis, stigma and its types, carers’ experiences of stigma, strategies to prevent or reduce stigma, coping skills and strategies to manage stress and feelings.	Education+ Life Stories+ Group Discussions
	Harris y Caporella. (2014)	U.S.A	Intergenerational Choir Provide opportunities for students, patients and carers to interact with university students, share experiences and provide an enjoyable social musical experience.	Contact + Intergenerational Interaction
General information about Alzheimer’s disease, treating and preventing Alzheimer’s disease in a dementia friendly community	Bray et al. (2015)	United Kingdom	Develop a toolkit of good volunteering practice to support volunteering opportunities that meet the diverse needs of BAME communities. Use of native language in awareness raising workshops. Cultural approach	Education with a cultural approach
	Bergeron et al. (2021)	U.S.A	Sharing knowledge about Alzheimer’s disease (warning signs, risk factors, prevalence data and differential causes of dementia, and tips for healthy brain ageing). The intervention group was divided into focus groups	Education + Group discussions
	Paone et al. (2015)	U.S.A	Dementia Friendly Communities Strategy. It cover the areas of: Screening and Quality Healthcare, Communities, Carers, Awareness, Government Policy Influence, Collective Action, Funding Infrastructure, Health Equity and National Visibility and Influence.	Education + Platforms + Health System + Governance
Programmes that have made use of digital platforms that are freely available	ADI & OPS. (2019)	Latin America and Caribbean	Talking about dementia helps fight stigma, normalises language It is a virtual platform. It provides data on mortality and morbidity of people with dementia in Latin America, has a test to assess awareness of warning signs, presents stories of people with dementia and their carers, and allows the use of promotional material.	Education + Audiovisual pieces + Life stories
	Isaacson et al. (2018)	U.S.A	Promotional strategy: use of Facebook.com. The impact of social networking to connect people for online training on Alzheimer’s disease.	Education through mass media
Supported programmes in the arts (museums, theatre, intergenerational choir)	Kontos et al. (2020)	Canada	Aesthetic immersion (Cracked.). Research-based theatre as a pedagogical strategy.	Education + Theatre + Life Stories
	Burns et al. (2018)	Australia	Influence of a theatre performance on the emotional affect of a general audience. Diversity of emotional experiences. Use of humour as a coping mechanism. Managing values such as honesty, optimism, creativity and poignancy.	Education + Theatre + Life Stories
	Au et al. (2020)	China	Perspective taking intervention (PT) in the context of intergenerational care Contents: monitoring mood and activities, planning pleasant events, monitoring behavioural responses, communicating with the care recipient, identifying sources of support and help-seeking skills. Monitoring behavioural responses, communicating with the care recipient, identifying sources of support and help-seeking skills.	Education + Intergenerational interaction
	Adams y Cotter. (2011)	U.S.A	It examines four ‘mini-case studies’ and museum installations that have developed programmes for people with dementia and their carers, all supported by MoMA’s Alzheimer’s Project, with activity-based, art-making, music, guided tours, creative storytelling and poetry, themed activities and theatre.	Education +Arts +Museums +Contact us
Multimedia campaign	Werner y Kermel. (2018)	Israel	Exposure to a multimedia campaign on stigmatising beliefs. The message and tone of the campaign refers to a socially active patient describing his daily routine. Guidelines for coping with memory loss	Education through mass media
Knowledge the disease and clinical aspects through culturally adapted bilingual presentations	Perales et al. (2020)	U.S.A	Culturally adapted dementia education programme Addressing ageing with dignity Changing attitudes towards dementia among Hispanic older adults	Education + Cultural adaptation
Awareness and knowledge of AD	Rosenzeweig. (2021)	U.S.A	Using vignettes to get participants to think about a fictional situation.	Education+ Life stories
	Montepare y Pandolfi. (2022)	United Kingdom	Dementia friendly environments. Educational interventions in higher education Knowledge about Alzheimer’s disease	Education + Curriculum

Note: This table presents the components of the research. It also presents the strategies.

Sixteen of the studies included an evaluation component to assess the effectiveness of the interventions in reducing social stigma towards Alzheimer’s disease; twelve of these included both pre- and post-programme measures, and three included multiple measures using different techniques to determine impact [29–31]. One study conducted a post-measurement [44], while the other six articles focused on reporting intervention development [4,32-35,45].

Of the total number of articles reviewed, only one reported follow-up after the programme and found that AD knowledge scores had improved but were in decline at the follow-up measurement two months after treatment [43]. Using pre and post measures, 10 studies reported an improvement in knowledge and attitudes towards people with Alzheimer’s disease. Key components of these studies emphasised the use of educational drama, sharing of experiences, use of vignettes and contact strategies. Three studies found no significant differences in knowledge and attitudes towards Alzheimer’s disease.

Due to the considerable heterogeneity of the interventions—regarding the general aspects of stigma addressed, the strategies employed (such as education, contact, life stories, and cultural and curricular adaptations in the case of university students), the outcome measures used, and the participant populations—the findings on the effectiveness of the interventions were varied.

Sixteen programs reported significant findings based on pre- and post-intervention measurements. Four studies reported changes in certain aspects of knowledge about Alzheimer’s disease; all of these shared a common emphasis on educational and contact-based strategies [38,39,43,46]. Additionally, the study by Perales et al. [47] also incorporated cultural adaptation strategies. The program developed by Kim et al. [38] reported statistically significant intragroup changes only, which may be attributed to the short duration of the intervention—just one week.

Two of the three programs focused on general information about Alzheimer’s disease, which employed mixed-methods approaches and had durations of two to three years, used strategies centered on education and the development of dementia-friendly communities supported by governance actions. These programs reported changes in stigmatizing attitudes and the sustained implementation of the interventions over time [32,33]. In the case of the study by Bergeron et al. [43], which included a control group and combined educational strategies with discussion groups, the findings suggested changes only at the intragroup level, with post-intervention measurements indicating stable scores over time.

Regarding emotional aspects and changes in stigmatizing attitudes [29,30,40,41], three studies reported significant improvements in stigmatizing attitudes, appropriate caregiving, knowledge of dementia, and the perceived impact of Alzheimer’s disease on individuals’ lives. Goldman & Trommer [29] and Au et al. [42] implemented strategies involving education, contact, and intergenerational interaction. The study by Chermahini [40] reported a reduction in perceived stigma at both intra- and intergroup levels, using a combination of educational strategies, life stories and discussion groups. Stites et al. [41] incorporated education and life story strategies to assess whether early detection of AD biomarkers could reduce stigma; however, the study did not find statistically significant changes in stigma scale scores following the intervention. Among the programs that focused on the use of digital platforms, one did not provide effectiveness data. The intervention proposed by the Pan American Health Organization [35] outlined guidelines based on education and the use of life stories, along with freely accessible media materials; however, it did not include implementation data. In contrast, the study by Isaacson et al. [45] provided data on the use of targeted Facebook advertising as an effective means of disseminating online education about Alzheimer’s disease. The study reported that this strategy successfully facilitated access to the program and yielded high satisfaction rates regarding both the content and program completion.

Three studies that incorporated the arts as a means to reduce stigmatizing attitudes reported significant changes in prejudice, emotional affect, and levels of satisfaction. Kontos et al. [31] followed a qualitative methodology and employed strategies such as education, life stories, and theater over a moderate duration of one year. Burns et al. [46], using a quantitative approach, implemented similar strategies. Although the program showed a significant effect, this finding should be interpreted with caution, as the researchers reported a loss of linkage between the pre- and post-performance questionnaires. As a result, they employed simulation techniques, such as sensitivity analysis, to address this limitation.

Harris & Caporella [30], who implemented contact and intergenerational interaction strategies over a program duration of more than two years, observed attitudinal changes among students, including greater understanding of dementia and the lived experience, reduced dementia-related stigma, and the development of meaningful social connections.

Finally, Adams & Cotter [34], in a technical report, used education and contact through a series of interactive sessions held in museums. Although the report did not aim to determine statistical significance, it provided qualitative insights into participants’ reflections throughout the process, as well as data on the number of activities that involved individuals living with Alzheimer’s disease.

The study that implemented a mass-access multimedia campaign reported significantly greater levels of positive emotions compared to negative ones following the intervention, using educational strategies aimed at the general public. However, exposure to the campaign was not significantly associated with any stigmatizing beliefs, either as a direct predictor or as a moderating variable of stigma toward Alzheimer’s disease [44].

Finally, it is noteworthy that in 17 programs, the primary outcome was a change in at least one dimension of stigma—specifically knowledge, prejudice, stigmatizing attitudes, or discrimination—particularly in those with longer durations. Although the quantitative studies varied in how they operationalized the outcome variables related to stigma reduction, the data collection procedures and instruments used to assess change over time were fairly consistent across studies.

Some studies were found to present a risk of selection bias due to high baseline levels of knowledge and low levels of stigma [38], attrition in one population group [41], the loss of first-year implementation reflections [32], and missing pre-intervention measurements [46]. Notably, the latter two studies still reported a significant positive impact.

## Discussion

A review of the findings indicates that North America remains the top country regarding public and structural anti-stigma intervention and management programs, with nine documents supporting this claim. The United Kingdom, Australia, and India each have two documents supporting their standing as leading proponents in this area. Latin America and the Caribbean rank lower in comparison. It is essential to acknowledge that the extent of these programs is contingent upon the macroeconomic policies of developed nations. As posited by Perea et al. [50] governments need genuine dedication to guarantee the right to health and support healthy lifestyles for at-risk populations.

Most interventions and programs for AD follow a biomedical approach, which focuses on diagnosing, treating, and controlling disease risk factors. However, this approach is lacking in educational programs to reduce the stigma of AD [16,51].

The literature review provides evidence that anti-stigma interventions targeting the general public, students, and communities—particularly through dementia-friendly environment strategies—can be effective in changing knowledge, prejudices, attitudes, and discriminatory behaviors toward individuals with Alzheimer’s disease across various population segments. As for the literature reviews, they only dealt with interventions to reduce the stigma of dementia in general and did not focus on Alzheimer’s disease, so they were excluded.

Another aspect to mention is that the programs with the most significant impact are those that address contact and psychoeducational interventions that promote dialogue, participation of the community and the actors involved in the care of AD [15,16,30], whose results have contributed to the reduction of isolation, the decrease in negative attitudes, the general reduction of stigma and social discomfort. Thus, the real and contextual needs of the socio-political and economic environment of the population, as well as the formal and informal support systems surrounding the person and his or her caregiver in the face of AD, should be considered first and foremost. As highlighted by Fuster [52], support and social ties act as a protector in the health of the individual in the physical and psychosocial or emotional consequences because it allows him to use these resources for the maintenance of physical and psychological integrity, which provides feedback about his own identity and performance in the prevention and care of his disease.

Another finding is that the use of social media, such as Facebook.com, can be a valuable tool for the dissemination of educational programs (conferences, symposia, webinars) for patients, especially in stage III, caregivers and the general public, where it has highlighted improvements in relationships and social connectedness of people with AD [44].

In Colombia, only the program “Communication Strategy for the Reduction of Stigma in Mental Health” was found [4], which includes the major neurocognitive disorders in Alzheimer’s disease, based on the Community Rehabilitation Strategy, which has two objectives: 1. “Participation and organization for social mobilization” and 2. The intervention focuses on representatives of institutions such as teachers, youth, business people and religious, but the guidelines for the different subtypes of dementia are not clear. In the specific case of Colombia, it was in 2018, through Resolution 488, that the country adopted its National Mental Health Policy, with an emphasis on positive mental health rather than illness. Currently, Colombia has a National Mental Health Policy for 2024–2033, which includes neurological disorders understood as medical conditions affecting the nervous system and also contributing to mental health disturbances. One potential factor contributing to the lack of implementation of stigma reduction programs is that, in middle-income countries like Colombia, between 40% and 70% of mental health spending is still allocated to psychiatric hospitals. Additionally, the country has yet to develop a national dementia plan [53].

The role that should be exercised by health and educational training institutions is to include within the academic curriculum interventions that address outside the aspects of AD, the health education strategies of anti-stigma programs. Likewise, it is important to consider that the professional (nurse, doctor, psychologist, social worker, among others) is really interested and committed to providing health education as an educational and health agent [52].

However, most of the studies or reports presented certain methodological limitations, such as focusing exclusively on increasing knowledge about Alzheimer’s disease, the absence of comparison groups, and the short duration of some interventions. Additional limitations included a lack of data on program implementation and small sample sizes. Moreover, the heterogeneity of the programs—in terms of content, implementation, outcome measures, and intervention strategies—highlights the need to interpret the findings on the effectiveness of stigma-reduction interventions with caution and in consideration of the specific context.

Among the limitations found in the studies is the lack of evaluation of the impact achieved in reducing stigma in the community and how these changes are maintained over time, both in the randomized controlled trials and in some mixed studies. The same happens in the programs developed by the Pan American Health Organization [35] and the campaign proposed by MinSalud [4] Some entities propose guidelines without providing data on program implementation. In this regard, Arthurton et al. [54] point out that although in 2017 the Member States of the World Health Organization (WHO) unanimously committed to adopting National Dementia Plans (NDPs)—a central goal of the Global Action Plan on the Public Health Response to Dementia—most countries remain far from achieving these objectives. Globally, only 20 countries have committed to developing an NDP. Among them, Brazil, the Dominican Republic, Panama, Puerto Rico, and Uruguay have plans currently under development, while Costa Rica, Chile, and Honduras have adopted a plan or strategy, but face challenges related to inadequate or absent funding. As a result, available resources tend to prioritize diagnosis, treatment, and care, while aspects related to dementia awareness and stigma reduction are often neglected.

The development and implementation of effective anti-stigma programs targeting healthcare professionals, caregivers, and patients with Alzheimer’s disease is of critical importance. First, although study findings are diverse, the development of culturally appropriate strategies tailored to specific groups—such as children and young people—could represent a promising initiative, given that public awareness of dementia campaigns tends to be quite low [4,18]. Therefore, while public initiatives have the advantage of reaching a broader audience, they do not necessarily result in sustained impact over time. In contrast, more targeted interventions may be maintained over the long term and produce specific changes in knowledge about Alzheimer’s disease and stigmatizing attitudes [30,31].

Second, participation in stigma-reduction programs could be made mandatory within the training curricula of healthcare professionals, who play a key role in both diagnosing patients and supporting individuals living with the disease, as well as their caregivers. This could contribute to reducing both structural stigma and caregiver-associated stigma. In contrast, a major limitation of public anti-stigma campaigns is their reliance on voluntary participation [38,41].

Digital and arts-based interventions, according to the analysis conducted, may contribute to positive changes in stigmatizing beliefs and help reduce social isolation, loss of dignity, and internalization of stereotypes. These types of programs tend to personalize the experience, positively impacting dignity through the use of language, supporting patients and caregivers in accessing self-care, and encouraging participant engagement. As such, they represent relevant community-based and educational tools to address stigma associated with Alzheimer’s disease [30,31,52].

Future research should prioritize follow-up evaluations that extend beyond pre- and post-intervention measurements. Given the dynamic, historical, and cultural nature of stigma, it is essential to design programs that reach populations through culturally adapted and personalized experiences aligned with the worldviews and characteristics of diverse social groups. In addition, interventions should be developed specifically for children and students, with the goal of breaking down myths and prejudices surrounding aging, old age, and dementia [51].

A major challenge for Latin America and the Caribbean is the development and effective implementation of public policies that not only focus on diagnosis and treatment, but also integrate stigma reduction into primary health care systems. Such policies should promote health education on dementia, raise awareness about the impact of stigma, and foster public engagement. It is also crucial to ensure adequate resources are allocated for the successful implementation of these initiatives [6,32,51].

Finally, it is important to mention that because we included a wide variety of studies (original articles, technical reports, and guidelines), we did not assess the risk of bias in the included studies. This may affect how we interpret the robustness of their results.

## Conclusions

Stigma towards Alzheimer’s disease can negatively affect access to accurate and timely diagnosis, effective utilization of health services and can lead to social isolation and depression [9]. Programs aimed at reducing the stigma of Alzheimer’s disease are essential to improve the quality of life of people with the disease and their caregivers in family and social settings [18].

As the goal of the study, programs and interventions to reduce social stigma toward Alzheimer’s disease focus on virtual strategies with programs that address knowledge and clinical aspects of the disease, and others are oriented toward strategies to raise awareness and improve the quality of life of people through the arts and to create dementia-friendly communities.

These programs and interventions have targeted the general public, school settings, communities, and minorities. However, there is a need to develop rigorous interventions in determining changes in stigma reduction and whether these are sustained over time.

## Supporting information

S1 Checklist Preferred reporting items for systematic reviews and meta-analyses extension for scoping reviews (PRISMA-ScR) checklist. (DOCX)
